# Climate change, planetary health and education in Africa – Some case studies and perspectives

**DOI:** 10.1016/j.onehlt.2026.101406

**Published:** 2026-04-24

**Authors:** Walter Leal Filho, Ilija Djekic, Newton Matandirotya, Felix Donkor, Umar Ibrahim, Richard Mbih, Adolphine Kateka, Jasmin Roeseler, Ayyoob Sharifi, Maria Alzira Pimenta Dinis

**Affiliations:** a School of Science and Environment, Manchester Metropolitan University, Street Manchester, Chester M1 5GD, UK; b Hamburg University of Applied Sciences, Hamburg, Germany; c Faculty of Agriculture, University of Belgrade, Nemanjina 6, Belgrade 11080, Zemun, Republic of Serbia; d Climate Change Adaptation and Resilience Centre, P.O. Box 5, Beitbridge, Zimbabwe; e Kgotso Development Trust, P.O. Box 5, Beitbridge, Zimbabwe; f Department of Geography Education, University of Education-, Winneba, Ghana; g Public and Environmental Health Department, Faculty of Basic Medical Sciences, Federal University Dutse, Ibrahim Aliyu Bye-Pass, Dutse, Jigawa State, Nigeria; h African Studies Program, The Pennsylvania State University, University Park, USA; i Department of Climate Change and Sustainable development, Global Water Partnership Tanzania, P.O Box 14200, Dar es Salaam, Tanzania; j The IDEC Institute & Network for Education and Research on Peace and Sustainability (NERPS), Hiroshima University, 1-5-1 Kagamiyama, Higashi Hiroshima City 739-8529, Hiroshima, Japan; k School of Architecture and Design, Lebanese American University, Beirut, Lebanon; l Fernando Pessoa Research, Innovation and Development Institute (FP-I3ID), University Fernando Pessoa (UFP), Praça 9 de Abril 349, Porto 4249-004, Portugal; m Marine and Environmental Sciences Centre (MARE), University of Coimbra, Edifício do Patronato, Rua da Matemática, 49, Coimbra 3004-517, Portugal

**Keywords:** Planetary health, Climate change, Climate education, Bibliometric analysis, Africa, Higher education

## Abstract

Africa is a diverse continent with a rich cultural heritage, abundant natural resources characterised by a great vulnerability to climate change and several health inequalities. These inequalities are also observed in the provision of planetary health instruction. This paper explores the interconnection between climate change and planetary health education inAfrican universities. Also,the study examines the extent to which some African universities are handling the theme of planetary health education. The method used involved a bibliometric analysis of climate change and health, focusing on specific institutions and initiatives that illustrate the extent of planetary health education carried out in Africa till date. The results of the study suggested that themes related to planetary health are increasingly being studied in African universities. Also, the study notes that the term “planetary health” is scarcely used in the reviewed literature; and to make planetary health a common topic in university programmes, the theme needs to be given a wider recognition and attention. The paper concludes by outlining measures that should be implemented as part of public health programmes to be offered at African universities, which may help in enhancing the adoption of education on planetary health.

## 1 Introduction

Climate change remains one of the biggest environmental-health threats to the human population worldwide, with estimates indicating an increase in the death rate of 250,000 per annum from climate related diseases over the years 2030–2050 [1]. Additionally, the population in sub-Saharan Africa is highly exposed to associated climate change health risks by a factor of three against other populations [2]. Climate change influences on the environment have manifested through variations in global mean temperatures and precipitation resulting in the high frequency extreme weather events [3]. These include droughts, hailstorms, devastating floods, heat waves, and forest fires, among others [4] exemplifying the vulnerabilities seen on the African continent [5]. The inter-tropical convergence zone, the El Niño—southern oscillation events, and the steady West African monsoon are climate drivers that further contribute to the vulnerability of the continent [6], [7].

Sub-Saharan Africa remains among the world regions bearing the highest toll from climate change, considerably increasing the existing inequality gap [8] moreso worsened by its limited resources and insufficient infrastructure that reduces both its adaptive and mitigatory capacity. The African region has displayed different levels of adaptation, with weather forecasting as an early warning system, monitoring programmes, education to raise awareness and additional public health systems infrastructure improvement [4]. Climate change has a substantial impact on the social determinants of health [9] constantly challenging the health sector's capacity. Environmental disasters such as flooding and cyclones directly affect the health sector by limiting access to care caused by breakdowns, disruption, and the impracticability of the system [10]. In parallel, high and extreme temperatures, including heatwaves, produce heat exhaustion and strokes, favouring severe health conditions and high death rates, overwhelming a system that is already relatively limited [11]. These challenges affect the mental health of the population already struggling with the burden of high poverty level as well as other socio-economic problems [12] thus threatening the planetary health (pH) and general well-being of the environment as communities turn to natural resources for survival.

The PH concept connotes addressing health threats attributed to anthropogenic climate change. It provides an integrative insight of nature, whereby humans and their health are considered as elements of nature, and individuals as key influences in the nature of interactions between society and the environment. Nevertheless, the PH concept is not integrated with educational practice [13]. PH is also regarded as an emerging discipline that shows the interconnections of human health with the well-being of our environment and surroundings [14]. Its focus is on introducing mechanisms for mitigating and adapting to the existing and future impacts of climate change [15], improving the integration of environmental health services and healthcare systems, as well as promoting sustainable changes to global health [16], [17], [18]. The Rockefeller Foundation-Lancet Commission on Planetary Health's report of 2015 defined planetary health as “the achievement of the highest attainable standard of health, well-being, and equity worldwide through judicious attention to the human systems - political, economic, and social - that shape the future of humanity and the earth's natural systems that define the safe environmental limits within which humanity can flourish” [11]. The same authors further consider the definition as the health of human civilization and the state of natural systems on which it depends on.

While PH has gained momentum in international discourse, its relevance and application within African contexts remain underexplored**.** Africa's high vulnerability to environmental stressors such as climate change, biodiversity loss, water scarcity, and food insecurity positions it as a critical region for advancing PH research and action [20], [21]. Studies have highlighted the intersection between ecological degradation and public health crises across the continent, calling for integrated, place-based responses informed by planetary health principles [22]). Despite this, climate change education frameworks in Africa often lack systematic incorporation of PH perspectives**.** Mugambiwa and Tirivangasi (2017) [23] argue that education remains reactive and fragmented, with limited emphasis on systems thinking or the socio-ecological dimensions of climate risks. Literature on regional nuances regarding climate change or planetary is limited. However, the West African region can enhance its resilience by building capacity in the domain of climate education and promoting it as a compulsory subject in the school curricula [24].

This calls for an appraisal of the approaches or methodologies adopted to optimise results [25]. Moreover, it is noteworthy that inclusivity as well as intergenerational justice are core themes in the concept of sustainable development which is threatened by climate change. However, [26] postulates that in general across the continent, there is limited youth engagement in decision-making processes which stifles novel solutions. This exacerbates the challenge of tokenistic youth participation in place of genuine involvement as vital stakeholders for climate action. Similarly, [27] note that while youth and community-level climate education initiatives have expanded, they rarely address the broader human-environment-health nexus. A small but growing body of literature calls for the localization of PH through culturally grounded and gender-responsive climate change education, which can empower communities to engage with sustainability challenges in contextually relevant ways [28]. However, current education and policy responses across most African countries continue to reflect a gap in interdisciplinary approaches, thereby limiting the transformative potential of PH to drive inclusive and resilient development.

Climate change also affects food- and water-borne disease incidences that are observed through the responsible factors, including heat-sensitive bacterial growth and floods spreading water contaminants, respectively [29]. Issues in food production and water scarcity are also visible, with underweight conditions among children in African countries like Kenya [30]) and Ethiopia [31]. Food security issues with disaster, bacterial growth, or warmer climate-associated factors have been associated with climate irregularities, impacting the distribution chain in African countries such as Nigeria [32] and Ghana [33]. Additionally, stressors such as poverty, family life, insecure employment, depression, social dysfunction, loss of confidence, and/or the fear of not being able to provide for the family are among the major causes of mental breakdown in the presence of natural calamity [34].

Consequently, concrete actions are needed as adaptation and mitigation approaches to respond to the fast-growing climate change crisis in African countries. According to the location of the hazard impacts, geography, the capacity to cope, public health responses can greatly differ within countries, with some better prepared than others [35]. Early warning systems to disseminate and communicate the importance of human health associated with planetary health through educational programmes can play a key role in raising awareness, building resilience, and improving preparedness [36], [37].

One of important mitigation measures is education and there is a necessity to target limitations and system weaknesses through the understanding of the current education programmes, performances, and their possible impact on behaviour for climate change mitigation [38]. The quality of education, highlighted in the 4th of the Sustainable Development Goals (SDGs) indicator framework, underlines the relevance of the nature of curricula in educational institutions [39]; [40], as well as UN SDG#13, target 13.3 – “Improve education, awareness-raising and human and institutional capacity on climate change mitigation, adaptation, impact reduction and early warning” (UN, SDG13).

The study's aim was to present an overview of planetary health education constraints in African universities and to explore the general understanding of climate change and health adaptation. On the African continent, pH education remains in its infancy [41]. The study is important because it sheds some light on the connections between climate change, pH, and education in the selected sample of institutions across the African continent.

## 2 Materials and methods

**Fig. 1:**
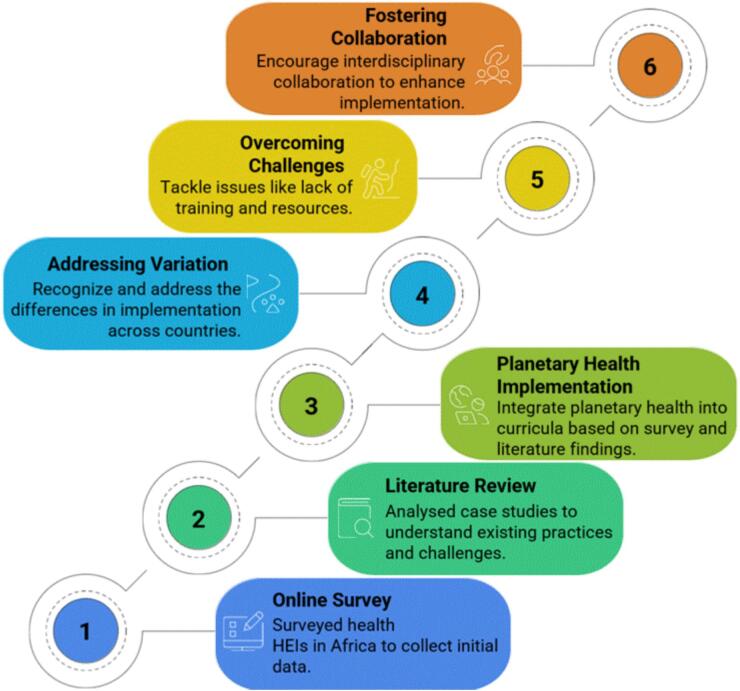
Study approach.

The study used a multi-method approach employing three distinct methodologies building upon each other, as illustrated in Fig. 1 which includes: a) a bibliometric analysis to explore current trends from literature; b) an online survey targeting key stakeholders in various African countries; and c) case studies focusing on selected African institutions and initiatives involved in climate change, planetary health, and education in Africa. The bibliometric analysis was done to identify key thematic focus areas in literature and potential gaps. Results of this analysis informed the design of the survey and case study selection. This combination of methods adds a degree of robustness to the study since it allows information to be collected from various sources, leading to a wide range of perspectives on matters related to PH at African universities.

The bibliometric analysis emphasizes the multiple social and environmental crises the African continent is facing, and a lack of sustainable practices and policy for dealing with them. Building on the results of the bibliometric analysis, an online survey was designed and disseminated among African public health education institutions. The online survey explored the integration of planetary health into curricula, as well as potential barriers for the integration. Lastly, two types of case study analyses were conducted to support the findings of the first two methodologies and offer good-practice examples. A selection of literature-based case studies reinforced the bibliometric analysis, by providing a detailed overview of some select studies on planetary health education in Africa.

### 2.1 Bibliometric analysis

To better unveil the intertwining between climate change, pH and education in Africa, the study conducted a bibliometric analysis. The input data for this analysis were bibliographic details of academic literature that were obtained from the online academic database SCOPUS (https://www.scopus.com/). Among various academic databases, SCOPUS was selected for its more comprehensive coverage of quality peer-reviewed research. To retrieve relevant studies, the study developed a search string that was informed by the relevancy of the literature [42]. The string included terms related to climate change and planetary health, universities and higher education institutions, health, and African countries. The detailed search string is as follows:

((“climate*” OR “global warming” OR “planetary health”) AND (“academic*” OR “university*”OR “higher education institution*” OR “HEI” OR “HEIs” OR “higher education” OR “college*”) AND (“health”) AND (“Nigeria” OR “Ethiopia” OR “Congo” OR “Egypt” OR “South Africa” OR “Tanzania” OR “Kenya” OR “Uganda” OR “Algeria” OR “Sudan” OR “Morocco” OR “Mozambique” OR “Ghana” OR “Angola” OR “Somalia” OR “Ivory Coast” OR “Madagascar” OR “Cameroon” OR “Burkina Faso” OR “Niger” OR “Malawi” OR “Zambia” OR “Mali” OR “Senegal” OR “Zimbabwe” OR “Chad” OR “Tunisia” OR “Guinea” OR “Rwanda” OR “Benin” OR “Burundi” OR “South Sudan” OR “Eritrea” OR “Sierra Leone” OR “Togo” OR “Libya” OR “Central African Republic” OR “Mauritania” OR “Congo” OR “Liberia” OR “Namibia” OR “Botswana” OR “Lesotho” OR “Gambia” OR “Gabon” OR “Guinea-Bissau” OR “Mauritius” OR “Equatorial Guinea” OR “Eswatini” OR “Djibouti” OR “R*union” OR “Comoros” OR “Western Sahara” OR “Cape Verde” OR “Mayotte” OR “S*o Tom* and Pr*ncipe” OR “Seychelles” OR “Africa*”))

The study focused on English-published articles between the years 2010 and 2021, with the initial search in the TITLE-ABS-KEY (titles, abstracts, keywords) field of the Scopus yielding a total of 395 articles that were further narrowed down to 327 in the final analysis after checking the abstracts and excluding articles that were not related to PH and education in Africa. Full bibliographic details of these documents were downloaded from SCOPUS for term co-occurrence analysis within VOSviewer. Among various software for bibliometric analysis, the study selected VOSviewer for its ability to provide detailed term co-occurrence maps using a user-friendly interface [43]. The term co-occurrence analysis of VOSviewer was used to understand the thematic focus and the overall focus on the research.

This analysis relies on text mining of the titles, abstracts, and keywords of academic articles and provides useful insights on thematic focus and interlinkages between terms and research clusters. The output of the term co-occurrence analysis is a graph network where each node represents a key term, and links indicate how terms are linked to each other. To systematise the results for further processing, the cut-off criterion was to consider words that were mentioned at least 10 times. If a term is mentioned fewer times it generates crowded map although a higher cut-off number may exclude important and relevant topics appearing less frequently [44]. It is recommended that, the minimum number of occurrences is at least 5, whereas 10+ have the potential of better visualization. Node size is proportional to the number of times a term has co-occurred with other terms, and link width is an indication of the strength of connection between nodes. Nodes that co-occur frequently and have strong links to each other form thematic clusters that are shown in separate colours [45].

### 2.2 Online survey of key stakeholders in selected African countries

The questionnaire survey was conducted between February 5th and September 23rd, 2022. The instrument (questionnaire) was made available using various platforms through a SoSci Survey link in the English language. The survey targeted experts and representatives of institutions of higher education, medical schools, and public health institutions from all 54 African countries.

The full survey tool can be found in Appendix 1. It was distributed via email across fast networks of the authors, the European School of Sustainability Science and Research as well as the Inter-University Sustainable Development Research Programme, both with many members from the African continent, employing a snowball sampling approach, encouraging participants to forward the link to relevant contacts. Before providing responses, participants provided informed consent to partake in the study, with all participants given an option to withdraw at any time. Every survey without the digitally acknowledged consent was discarded from the final data set. The remaining feedback covered 10 African countries, including Ghana, Nigeria, Cameroon, Zimbabwe, South Africa, Egypt, Sudan, Kenya, Tanzania, and Madagascar. The obtained dataset was uploaded on an Excel sheet, transformed, and coded before analysis. The data was then disaggregated per country and analysed for frequencies. Data from open qualitative questions was analysed for content and quantified in order to enable comparison.

### 2.3 Case studies on health education in Africa

The study also used a case studies approach. The choice of case studies focusing on planetary health in Africa is driven by the continent's unique socio-ecological challenges and its rich biodiversity. Each case study highlights the intricate connections between human health and climate resilience. By examining diverse settings across various African regions, a holistic understanding of how environmental factors, such as land use changes, pollution, and climate change, impact human health, is provided. This comprehensive approach enables the identification of effective strategies for mitigating health risks and promoting sustainable practices, fostering local engagement, and informing policy interventions tailored to Africa's specific needs and contexts.

Data were collected from several sources, including grey literature, considered where relevant, reports, documents, as well as information available from the official websites of HEIs and medical institutions in Africa. A total of case studies focusing on planetary health in Africa were identified. Each case was analysed as a unique entity, aiming to address the following aspects: i) relevance to planetary health, focusing on addressing planetary health challenges and integrating environmental sustainability into health professions curricula; ii) geographical representation, including case studies from different regions of Africa to capture diverse perspectives and experiences; iii) engagement with local solutions, with an emphasis on initiatives, training programmes, and adaptation measures developed within African communities to address climate change and health challenges; iv) alignment with policy objectives, considering case studies that align with regional policy frameworks and priorities for climate change adaptation and health promotion; v) interdisciplinary approach, incorporating studies that explore the intersection of health education, environmental sustainability, and climate change resilience in Africa; vi) evidence-based insights, selecting case studies backed by scientific research and peer-reviewed publications to ensure credibility and reliability of information, and finally, vii) potential for scalability and impact, assessing case studies with potential for scalability and replication in other African contexts to promote a broader adoption of effective strategies for planetary health education.

The case studies were also selected based on literature-based reported initiatives, so they are duly documented and can be verified. Overall, the selected case studies represent a diverse range of planetary health challenges, ensuring geographical representation, scientific rigor, and applicability to African contexts. They also provide evidence-based insights and scalable solutions while emphasising local engagement and interdisciplinary approaches.

## 3 Results

In this section we present the results from the bibliometric analysis, the online survey and case studies, which are presented in turn.

### 3.1 Term co-occurrence from bibliometric analysis

**Fig. 2:**
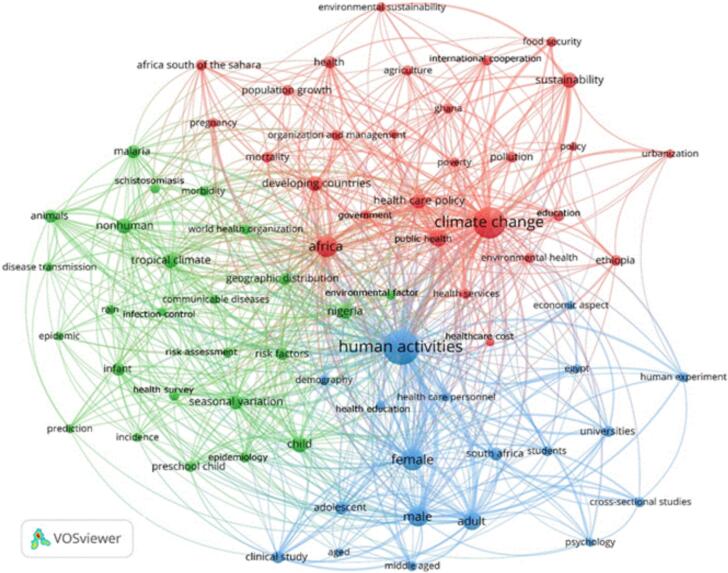
The output of the term co-occurrence analysis.

The results of the term co-occurrence analysis are shown in Fig. 2. The analysis shows 72 key terms and the way they are linked to each other. Three key thematic clusters were identified and presented in different colours capturing information from the title of the selected paper, key words, highlights and abstracts. These clusters cover multiple issues related to the social and environmental determinants of health. The strong connections between different terms and clusters indicate that addressing the intricate health challenges necessitates a comprehensive interdisciplinary strategy that effectively considers ecological, social, and economic factors in preventing and controlling the spread of diseases. However, the analysis highlights limited representation of the term “planetary health” in the bibliographic data, pointing to an area requiring further academic integration. Better recognition and promotion of this concept could provide opportunities for more integrated approaches toward health.

The **red** cluster dominates the figure, focusing primarily on social and institutional issues such as poverty, urbanisation, healthcare services and costs, education, food security, organisation and management, and international cooperation. Africa faces significant challenges due to the complex interconnection between rapid urbanisation, population growth, poverty, pollution, food security, environmental health, and sustainability [42].

Cities struggle to provide essential services and infrastructure as urbanisation outpaces their capacity, leading to informal development and increased poverty and pollution. Urban expansion often encroaches on arable land, threatening food security and environmental well-being [46]. The lack of sustainable practices in rapidly growing urban areas exacerbates climate change impacts, resulting in more severe weather events that further jeopardize food production and public health [47]. These growth patterns lock cities into unsustainable futures and create emission-intensive urban forms. This reflects the complex intersections between urbanisation, health, and sustainability observed in the African literature cluster. The **green** cluster focuses on non-human factors, communicable diseases, and issues related to epidemiology and infection control. In Africa, the landscape of epidemics and infectious diseases is undergoing significant changes due to rapid urbanisation, climate change, and other socio-economic factors.

These changes are intricately linked to planetary health and education, creating a complex web of interactions that demand comprehensive analysis and intervention. Climate change is a critical driver of disease patterns in Africa. Variations in temperature and rainfall throughout the seasons have a profound impact on the spread of vector-borne diseases like malaria, particularly during the rainy season when mosquito populations peak [48]. Rising temperatures are expanding the geographical range of disease vectors, leading to the emergence of diseases in previously unaffected areas. This changing landscape reflects the increasing vulnerability of African populations to shifting disease vectors influenced by climate change.

The geographical spread of diseases is altering as certain areas experience a rise in prevalence due to environmental changes favouring disease vectors [49]. This phenomenon highlights the need for targeted education initiatives that address region-specific health risks and promote sustainable practices to mitigate the impact of climate change on disease transmission. International cooperation plays a crucial role in addressing these challenges by providing technical expertise and financial backing to implement disease control programmes. However, the effectiveness of these programmes is contingent on their integration with local education systems and cultural contexts. Enhancing education capacities forms a critical component of a multi-faceted approach to managing communicable diseases in Africa, aiming to reduce their incidence for a healthier future.

The **blue** cluster focuses on universities, health education, and healthcare personnel, emphasising the pivotal role of higher education institutions in addressing the complex health challenges caused by rapid urbanisation, climate change, and communicable diseases in Africa [50]. Universities serve as hubs for research, innovation, and knowledge dissemination, bridging the gap between scientific understanding and practical application in the context of planetary health. By conducting rigorous cross-sectional studies, healthcare personnel and researchers at universities offer valuable insights into the epidemiology of diseases affecting diverse demographic groups across all ages [51]. These studies contribute to a deeper understanding of health issues among different populations and inform the development of targeted interventions that consider the interplay between climate change, environmental factors, and human health.

The knowledge gained from such research informs health education curricula, preparing future healthcare professionals with the necessary skills to deliver effective care based on evidence-based interventions. This education must encompass not only traditional medical knowledge but also an understanding of the complex interactions between climate change, planetary health, and human well-being. Integrating concepts of environmental sustainability, climate resilience, and eco-health into medical and public health curricula is essential for developing a workforce capable of addressing the evolving health challenges in Africa. Thematic clustering revealed significant terms linked to planetary health education, yet showed limited prominence of the term ‘planetary health’ itself. The bibliographic analysis highlights the role of higher education institutions in linking research, education, and public health adaptation strategies.

### 3.2 Online survey of key stakeholders in African countries

A total of 50 representatives from 10 African countries participated in the survey. Respondents were affiliated with a range of public health academic institutions, research centres, and ministries, as well as public organisations such as universities and non-governmental organisations (NGOs). The participating institutions comprised schools of public health (25%), public health research centres (25%), and other organisations (50%)—including ministries (such as Agriculture), state universities, NGOs, and additional research bodies (Table 1).

**Table 1:** Type of participating organisations.

Organisation type	Relative Frequency (%)
School of Public Health	25
Public Health Research Centre	25
Other institutions (NGOs, government ministries, community-based organisations)	50
Total	**100**

Respondents identified inclusion in academic curricula (39%), community sensitisation and awareness-raising (21%), and targeted education initiatives (18%) as the primary strategies for expanding the uptake of planetary health and enhancing knowledge across Africa. Less frequently cited approaches—each selected by fewer than 10% of participants—included staff training, dedicated funding, theoretical instruction, institutional collaboration, and government support (Table 2).

**Table 2:** Ways of increasing knowledge of planetary health.

Suggested way of increasing knowledge on planetary health	Relative Frequency (%)
Inclusion in curriculum	39
Sensitisation and community awareness	21
Targeted education	18
Staff education	9
Theoretical education	7
Collaboration and governmental support	6
Total	**100**

**Fig. 3:**
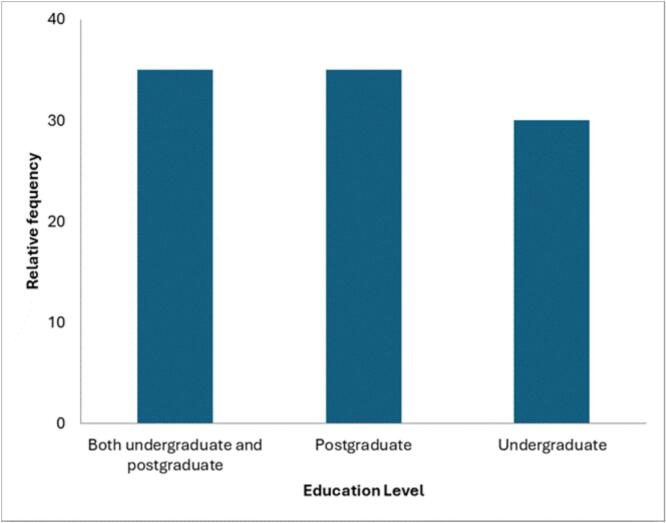
Level at which planetary health education is being delivered in Africa.

As shown in Fig. 3, 80% of respondents regarded targeted planetary health education as essential for preventing and managing emerging challenges linked to climate change, including pandemics and disease outbreaks. Over 70% believed that such education is vital for producing well-trained professionals. When it comes to programme implementation, 30% reported that planetary health is taught only at undergraduate level, 35% indicated it is offered exclusively at postgraduate level, and the remaining 35% confirmed that modules are integrated across both study levels within their educational institutions.

**Fig. 4:**
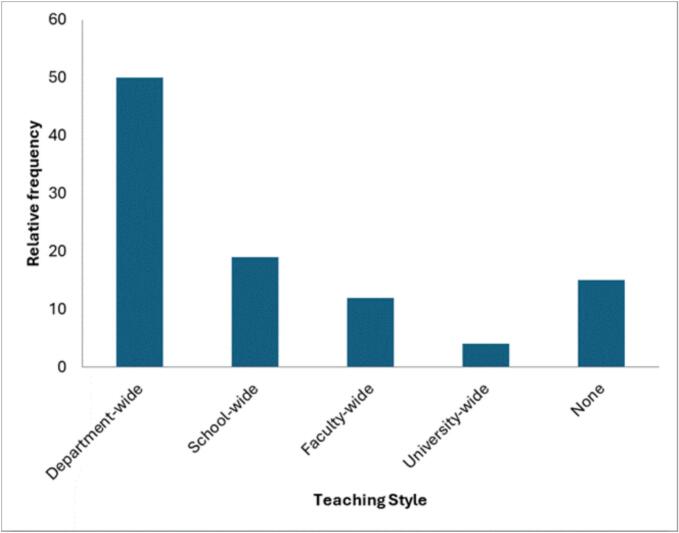
Planetary health course teaching style in Africa.

As illustrated in Fig. 4**,** reported teaching approaches varied, with 50% of respondents indicating department-wide delivery, followed by school-wide (19%), faculty-wide (12%), and university-wide implementation (4%). Additionally, 15% of respondents did not specify a teaching format.

According to the country of origin, respondents described the institution's emphasis on planetary health to be null (Cameroon, Kenya), limited (Kenya, Zimbabwe), moderate (Madagascar, Kenya, Nigeria, Cameroon), significant (Tanzania, South Africa, Kenya, Nigeria) or very significant (South Africa, Sudan, Tanzania, Ghana, Nigeria, Kenya). In most of the respondents' organisations, there is an intention to include planetary health education in the coming year.

**Fig. 5:**
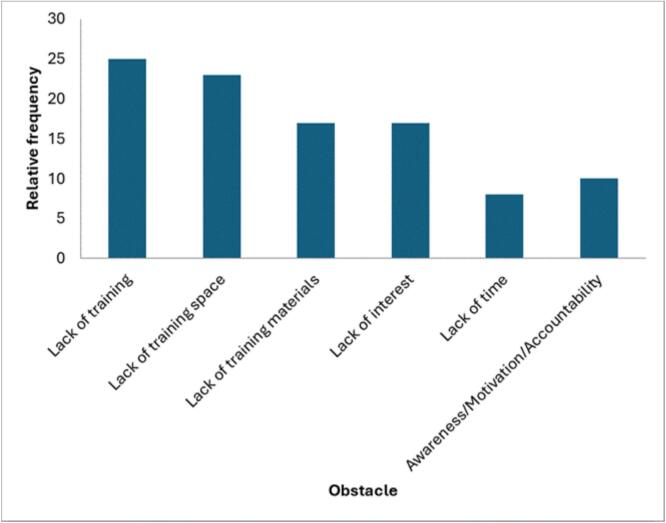
Obstacles to teaching planetary health education in Africa.

Respondents frequently cited curriculum integration and targeted education as central measures. Institutional and logistical barriers such as lack of training, space, and materials were also commonly reported (see Fig. 5). The most common obstacles included lack of training (25%), lack of space (23%), lack of materials (17%), lack of interest (17%), and lack of time (8%). Additional challenges cited were limited awareness, insufficient motivation, and lack of institutional accountability. When disaggregated by country, specific barriers varied: lack of space was predominant in Egypt; time constraints were highlighted in Kenya; both space and time limitations were reported in Nigeria; and in Tanzania, lack of space and teaching materials emerged as the main obstacles.

Overall, the survey findings corroborate the bibliometric analysis by showing that PH education is still limited in scope and unevenly integrated across institutions in Africa. While awareness is increasing, respondents highlighted curricular inclusion and targeted education as key entry points, though implementation is hindered by barriers such as lack of training, resources, and institutional support.

### 3.3 Case studies

Table 3 presents a collection of institutions actively addressing planetary health challenges across Africa. These organisations, among several others that could have been also included, play a crucial role in integrating planetary health into professional health education by developing research-driven solutions, implementing educational programs, and fostering interdisciplinary collaborations. Several institutions, such as The African Population and Health Research Centre (APHRC) and The African Institute for Development Policy (AFIDEP), contribute to planetary health by generating evidence-based research and policy recommendations that inform educational curricula and public health strategies. Their work ensures that planetary health education is grounded in scientific rigor and policy alignment, fulfilling a critical need for informed decision-making in health and environmental governance. Additionally, institutions such as the South African Academy of Family Physicians and the West African Academy of Public Health focus on integrating planetary health principles into medical and public health training.

**Table 3:** African-based institution case studies on planetary health.

Institution	Mandate	Reference
The African Population and Health Research Center	Aims at generating evidence-based research, strengthening research capacity, and engaging in policy to inform action on population health and well-being in Africa.	[52]
Planetary Health Eastern Africa Hub	Offers opportunities to learn and share about planetary health and the planetary health emergency in Eastern Africa by stimulating regional community building, providing education, and encouraging policy-making to face current planetary health emergencies.	[53]
The African Institute for Development Policy	Aims at addressing climate change and planetary health challenges in Sub-Saharan Africa through evidence-based research projects that address the health impacts of climate change	[54]
West African Institute of Public Health	Works on achieving an optimum public health system in the region, as it is necessary to meet the shortfall of health professionals across the countries in the region, as well as to strengthen the knowledge base around the implementation of science and research	[55]
Madagascar Health and Environmental Research	Its research agenda is based on the intersection of human health and the environment. The institution is also investigating the effects of environmental change on human health.	[56]

These institutions emphasize the development of curricula that prepare health professionals to address climate change-related health risks, thereby aligning with the need for workforce capacity-building in planetary health. Similarly, the Planetary Health Eastern Africa Hub fosters regional knowledge-sharing and policy engagement, highlighting the importance of a collaborative approach in strengthening planetary health education. Furthermore, some organisations, such as the Community and Family Aid Foundation and the Africa One Health Network, extend planetary health education beyond formal academic settings by engaging communities and promoting grassroots solutions. Their focus on social determinants of health and climate resilience ensures that planetary health education reaches diverse stakeholders, including policymakers, educators, and local communities. Globally, the institutions presented in Table 3 illustrate a multi-faceted approach to planetary health education in Africa, bridging gaps between research, policy, professional training, and community engagement. Their collective efforts reinforce the necessity of a transdisciplinary and regionally relevant planetary health framework that integrates environmental sustainability, public health, and climate adaptation strategies within African educational and healthcare systems.

The case studies on health education highlighted in Africa (Table 4) underscore the urgent need to integrate PH and environmental sustainability into the curricula of health professions. Additionally, the studies reveal pressing environmental challenges in Africa, emphasising the need to address issues such as conflict, food insecurity, and institutional fragmentation to enhance adaptive capacity to climate change. There is a call for integrating climate change considerations into developmental frameworks and fostering collaboration between stakeholders. Moreover, exploring strategies like agroforestry and advocating for climate justice are crucial aspects that deserve further attention to address health issues in African communities.

**Table 4:** Literature-based representative case studies on planetary health education in Africa.

Title	Study objective/s	Key findings	Reference
Addressing planetary health challenges in Africa	The study explores some of the core planetary health challenges facing Africa and response measures	The study is premised on the 21st Conference of Parties (COP21) fringe forum, “Healthy Lives on a Healthy Planet”. It throws the spotlight on present and future trajectories of some core environmental challenges in Africa and their impacts on health and well-being.	[17]
Adapting to a changing environment: inspiration for planetary health from East African communities	The study investigates how Africa's vulnerability affects its planetary health	The study established that background factors such as conflict, food insecurity, and displacement undermine the continent's adaptive capacity to climate change. Authors observe that poorly resourced and fragmented institutional frameworks on the continent also compromise robust policy execution. Nevertheless, these have engendered a number of bottom-up or tailored grassroots solutions which are often overlooked.	[58]
Making a case for planetary health in sub-Saharan Africa. Policy Brief	Prevailing climate change measures fail to address climate change issues as policy frameworks do not deal with the cross-cutting health effects of climate change.	The findings in this study were that mainstreaming climate change within developmental frameworks is compromised owing to the absence of regular vulnerability and adaptation reviews. This necessitates creating health impact, vulnerability, and adaptation indicators to facilitate monitoring as well as make the process more iterative. Authors indicate that climate adaptation and mitigation measures are largely working in silos, which undermines their effectiveness due to poor collaboration between relevant stakeholder institutions.	[57]
A Planetary Health Perspective on Agroforestry in Sub-Saharan Africa	The study explores the consequences of the changes in tree-based farming (agroforestry) and land, on food security, disease spread and migration in Sub-Saharan Africa.	The study asserts that agroforestry can help mitigate some of the core health issues plaguing African communities. Authors thus investigate some of the underlying determinants of agroforestry uptake, looking at some drivers of social and environmental change.	[59]
Achieving climate justice, safeguarding planetary health: Diagnosis and demands from next generation leaders for COP27 and beyond	This case study argues for climate justice and health to be centred on planetary health interventions and debates.	The study postulates that the challenge with climate change and planetary health can be traced to colonialism as underlined in the recent briefing of the United Nations Intergovernmental Panel on Climate Change (UN IPCC. In the view of the authors, the prevailing capitalism prioritises exploitation to the detriment of the planet and indigenous communities who bear the brunt of the climate crisis. The legacy of colonialism is evident in the climate negotiations, as the interests of the rich hold sway over the concerns of Indigenous communities.	[60]

Additionally, the findings from [57], [58] highlight the inadequacy of current climate change policies in addressing health impacts effectively. These studies align with the selection criteria as they emphasize engagement with local solutions and the need for policy alignment. Otieno et al. (2022) [58] particularly focuses on how conflict, food insecurity, and displacement weaken Africa's adaptive capacity, underlining the necessity for robust and context-specific interventions. In addition, the study from [59] explores the potential of agroforestry in mitigating health issues in African communities.

The study analyses the drivers of agroforestry uptake and its implications for food security, disease spread, and migration in Sub-Saharan Africa. Its inclusion is justified by its interdisciplinary approach, integrating environmental sustainability and public health, and its potential for scalability as a nature-based solution to planetary health challenges. Guinto et al. (2022) [60] provide a critical lens on climate justice, demonstrating how historical inequalities continue to shape Africa's vulnerability to climate change. Their arguments resonate with the broader discourse on PH, highlighting the need for structural changes at global and regional levels. This case study was selected as it aligns with the policy objectives criterion and offers a framework for advocating equity-driven solutions.

Taken together, the case studies provide qualitative depth to the bibliometric and survey findings, illustrating how selected African institutions are beginning to integrate planetary health into their education and research missions. They also demonstrate practical challenges and opportunities, reinforcing the general trends identified in the other methods.

## 4 Discussion

The study explored the current trends and status of climate change and PH education within a educational institutions that are on the continent of Africa. In addition to population growth, the poor management of Africa's natural resources and ecosystem, the exponential urbanisation, and the increased risk for climate change are exacerbating the situation [16]). This far PH education has gained ground globally and across disciplines and institutions in Africa after the launch of the 2015 Rockefeller Foundation-Lancet Commission on Planetary Health [62], and since then there has been an emergence of researchers on the continent with a focus on PH. Embracing a PH perspective that acknowledges the interconnectedness of human, animal, and environmental health is essential for promoting health [63].

Additionally, the study highlights a fragmented but emerging research landscape within the field of PH and climate change. Although health and climate change dominate African scholarship, the marginal presence of ‘planetary health’ as a term suggests that the concept has not yet gained traction as a unifying academic framework. This lack of explicit terminology may reflect both structural and disciplinary silos, limiting the potential for integrated planetary health curricula. The survey responses confirm that these bibliometric gaps are mirrored in institutional practice. Despite growing awareness, planetary health education remains unevenly implemented across institutions. Respondents pointed to curricular inclusion and awareness-raising as priorities, yet repeatedly cited structural barriers—such as lack of resources and staff training—that prevent systematic uptake.

Meanwhile, education, healthcare policies, and international cooperation are crucial in addressing the challenges of rapid urbanisation, population growth, and environmental sustainability in Africa [64]). Education is important for equipping individuals with the knowledge and skills to make well-informed decisions concerning their health and environment. It can enhance agricultural practices, promote understanding of sustainable development, and raise awareness about public health issues. Accessible healthcare policies and services are essential for improving maternal and child health, reducing mortality rates, and addressing the impact of pollution on health as well as climate change. By investing in healthcare infrastructure and ensuring access to quality services, African countries can improve overall public health outcomes [65]. Addressing the urbanisation-related health challenges described in the bibliographic analysis calls for a comprehensive strategy for urban planning and policy-making. Such strategies must consider interactions across multiple dimensions of resilience and sustainability [48], particularly in African cities where vulnerabilities are deeply interwoven.

International cooperation plays a crucial role in sharing knowledge, resources, and technology to facilitate the implementation of best practices in urban planning while also supporting climate change mitigation efforts, effectively contributing to robust health systems' development amidst direct/indirect impacts of urbanisation on food security/environmental/public concerns [67]. These elements work together to create a synergistic effect that improves public health, reduces mortality and pregnancy-related issues, and strengthens communities against the adverse effects of rapid urbanisation and climate change. Education and healthcare form the foundation of a healthy society, which can transform challenges into opportunities for sustainable growth and development.

The increasing environmental and health challenges on the African continent, including extreme weather events and COVID-19, highlight the interconnectedness of global environmental, social, and health crises as well as the indications and importance of planetary health. This shifting disease landscape necessitates adaptive education strategies to prepare communities for new and re-emerging health challenges influenced by climate variability. Currently, the knowledge of planetary health education in Africa exists in isolated forms in various institutions; however, an increasing number of academic institutions in Africa today are engaged in and promoting planetary health research within their different academic disciplines. It needs to be emphasized that higher education in Africa is facing several challenges in terms of student enrolment, curriculum quality and research focus [68]. This can be observed in disciplines like geosciences, health (human and veterinary medicine), family and community development, humanities, and the social sciences, providing the basis for the emergence of a multidisciplinary domain of planetary health to advance a better approach to addressing current global environmental and health challenges [68].

The findings align with UN SDG Target 13.3, which advocates for enhanced climate change education and awareness (https://sdgs.un.org/). Yet, implementation barriers persist. Educators face challenges in pedagogical capacity and material resources, while students—often from Generation Z—expect interactive and values-driven instruction. This highlights the need for extrospective, societal, and personal-processual pedagogical approaches [69] to equip learners with relevant sustainability competencies.

The rapid growth in socio-economic, environmental, and health challenges in Africa as shown by an increase in rainfall variability, food insecurity, urbanisation, floods, droughts, malnutrition, biodiversity loss, and pollution are indications of the urgent need for PH education in the region [16], [70]. In exploring the main planetary health challenges in Africa and how such challenges may be addressed, [16] identified high population growth rates, urbanisation, natural resources, and ecosystem management as the three main drivers of environment-related challenges, whereby an African response could make a difference in limiting Africa's exposure in the long term. Despite its urgent need, the concept of planetary health is still new in Africa, and most African academic institutions have yet to accept and incorporate planetary health as an emerging multidisciplinary domain into their curriculum [70].

Most PH programmes across Africa are still at the infancy stages however face a challenge of lacking appropriate funding for intended projects. Some of these, including the Planetary Health Eastern African Hub, focus on the eastern African community and partner with other international organisations like Women Leaders for Planetary Health, Planetary Health Academy, and Planetary Health Alliance in its projects [71]. It needs to be emphasized that the main obstacle in innovating higher education with climate change issues is the competence of teaching staff and lack of funding resources for climate change research [50]. In parallel, there is a need for improving overall pedagogical approaches to achieve adequate sustainability competences of the new generation [72]. It is important to provide students with transdisciplinary skillsets to make societies more sustainable [71]. It should be considered that the students are a generation born between 1995 and 2010 (aka Gen “Z”), that makes up almost a third of the world's population being the largest global consumer group born in the digital age with specific perceptions, behaviours and preferences [73].

Current lecturing does not develop necessary sustainability competences outlining three pedagogical approaches needed in higher education: extrospective-social, cogitative-societal and personal-processual [69]. PH education will provide multidisciplinary problem-solving measures for the increasing environmental and health challenges in Africa. It will further create opportunities for raising awareness, knowledge exchange, and connections among the various generations of Africans within communities and institutions across the region for a healthier future. In contrast to African institutions, universities in Europe and North America have benefitted from earlier policy integration and sustained funding structures. For instance, in Germany and the UK, planetary health learning objectives have been embedded within national accreditation frameworks, facilitating mainstream curriculum inclusion [74].

This study has some limitations. Firstly, the bibliometric analysis focused on a set of specific terms and did not cover related aspects such as socio-economic aspects and financing. The bibliometric was only focused on English publications as the predominant language associated with scientific publications, and did not consider other languages such as French or Portuguese. Further, the reviewed literature mainly provides descriptive information on the interlinkages between planetary health and climate change education. This limited our ability to provide quantitative details on the dynamics of this nexus. Secondly, whereas the online survey identified some current trends, the variability of regional contexts across Africa can complicate generalisations, as cultural, economic, and even environmental factors significantly influence the ways planetary health issues are tackled. Thirdly, the study might not fully address the interdisciplinary nature of planetary health, overlooking critical collaborations between fields such as medicine, environmental science, and policy. Despite these constraints, the study provides a welcome addition to the literature since it provides an overview of the current situation in relation to PH education in Africa today.

## 5 Policy implications

Climate change education can work as a persuasive tool at community levels [75] particularly when infused with participatory approaches. For an effective integration however, initiation targeting lower academic levels first will be fundamental, inspiring stewardship and environmental care at a young age [76]. Community empowerment strategies for a better understanding of the science of the changing climate can be both formal and informal [75]. Diverse factors are still being reported that considerably hinder the establishment of climate change education at academic levels [50].

The integration of climate change curricula within existing courses that are compulsory is therefore seen as a need that would readily solve this challenge [77]. Other avenues to advance climate change education include short and long non-formal training that can be customised and tailored to context.

Universities are key centres for innovation, developing new strategies for infection control that consider the unique needs of each demographic group within the context of a changing climate. This includes research into climate-resilient health systems, sustainable urban planning to reduce disease transmission, and the development of early warning systems for climate-sensitive diseases. Creating an environment focused on learning and discovery allows universities to shape resilient healthcare policies capable of enduring environmental changes and societal pressures Universities also play a crucial role in fostering interdisciplinary collaboration, bringing together experts in climate science, public health, urban planning, and social sciences to develop holistic solutions to the complex challenges facing Africa.

Moreover, universities can serve as catalysts for community engagement and education, extending their reach beyond the campus to promote health literacy, environmental awareness, and sustainable practices among the broader population. Such outreach approaches are crucial for building community resilience to the health impacts of climate change and fostering a sense of collective responsibility for PH. Overall, the interactions between climate change, planetary health, and education in Africa are multifaceted and dynamic. Addressing such dynamics requires a comprehensive approach that integrates cutting-edge research, innovative education strategies, and community engagement. By strengthening the role of universities and enhancing international cooperation, Africa can develop robust, climate-resilient health systems that protect and promote the well-being of its populations in the face of ongoing environmental changes.

There is a growing need to enable health professionals to protect public health in relation to climate change challenges through improved education in Africa, for instance, by directly integrating concepts of planetary health into curricula. One approach could be the alignment with education for sustainable healthcare objectives outlined by the Association for Medical Education in Europe [81]. Therefore, the perspective and strategies undertaken by higher education and public health institutions, in the mission to foster adaptation and mitigation actions as well as a better understanding, will contribute to shaping curricula, making education a crucial instrument.

The study established that planetary education teaching is still very scant and limited in scale across Africa, as most institutions are offering (such) them as optional courses. This gap can be reduced by integrating programmes and courses in tertiary institutions to increase climate and planetary health education reach. The study further noted that the term “planetary health” is scarcely reflected in the reviewed literature. The study shows that several institutions throughout Africa work on increasing planetary health education through various projects and by organising different training and awareness actions. As the continent grapples with the impacts of a changing climate — including extreme weather events, declining biodiversity, and health disparities —the role of education emerges as a critical catalyst for building resilience and fostering transformative change.

The approaches or methodologies employed in climate education need to capture the multidimensional nature of climate change by coupling it with planetary health education whilst integrating the scientific dimension with socio-economic and political context. Additionally, whilst the impacts of climate-induced changes are often global in nature, the particular threats and requisite responses are largely regionally or spatially specific.

## 6 Conclusions

Africa's long-term sustainable development is threatened by climate change with implications for implementing the UN SDGs and related “Leave No One Behind” agenda. Climate change education can serve as a remediation vehicle for addressing climate-induced challenges in Africa when coupled with planetary health to help address its multidimensional effects. This study contributes knowledge that will help inform policy on effective climate change education and is of relevance to policy makers, researchers, NGO's, advocacy groups and practices. It feeds into the African Union's Climate Change and Resilient Development Strategy and Action Plan (2022−2032) and SDG4. Future research may focus on analysing the effects of existing measures taken in different African countries regarding planetary health and education in a climate-changing environment, specifically integrating multiple databases, such as PubMed, for a more comprehensive bibliometric analysis.

## CRediT authorship contribution statement

**Walter Leal Filho:** Writing – review & editing, Writing – original draft, Methodology, Investigation, Formal analysis, Conceptualization. **Ilija Djekic:** Writing – review & editing, Writing – original draft, Visualization. **Newton Matandirotya:** Writing – review & editing, Writing – original draft, Methodology, Investigation. **Felix Donkor:** Writing – review & editing, Writing – original draft. **Umar Ibrahim:** Writing – review & editing, Writing – original draft. **Richard Mbih:** Writing – review & editing, Writing – original draft. **Adolphine Kateka:** Writing – review & editing, Writing – original draft. **Jasmin Roeseler:** Writing – review & editing, Writing – original draft. **Ayyoob Sharifi:** Writing – review & editing, Writing – original draft. **Maria Alzira Pimenta Dinis:** Writing – review & editing, Writing – original draft.

## Declaration of competing interest

The authors declare that they have no known competing financial interests or personal relationships that could have appeared to influence the work reported in this paper.

## Data Availability

Data will be made available on request.

## Appendix A Appendix 1. Survey tool

Climate Change, Planetary Health and Education in Africa: an overview of trends.

### A.1 Please choose your country from the list

[MD2]

1. Is your organisation

(a) A School of Public Health

(b) A Department of Public Health or similar

(c) A Research Centre on Public Health

(d) Others (please specify)…………………………………….

3. To which extent does your School (or Department/research centre) give an emphasis to planetary health as part of your health education programmes?

(a) A very significant emphasis

(b) A significant emphasis

(c) A moderate emphasis

(d) A little emphasis

(e) No emphasis (If so, please go straight to Question 9)

4. For how long has planetary health as a topic been part of your teaching programme?

(a) 1 year or less

(b) 2–5 years

(c) 6–10 years

(d) 11–15 years

(e) Over 15 years

5. At which levels are planetary health modules being taught?

(a) Undergraduate level

(b) Postgraduate level

(c) Both

6. How are planetary health modules predominantly taught? (Please tick one option)

(a) University wide courses

(b) Faculty wide courses

(c) School wide courses

(d) Department wide courses

7. Do you regard planetary health as an important component of your programme in producing holistic practitioners?

(a) Yes

(b) No

8. Do you consider planetary health education as a tool that can be used to prevent and manage future pandemic outbreaks?

(a) Yes

(b) No

(if e was selected in question 3)

9. What are the barriers that prevent the integration of planetary health as part of your education programme? (multiple answers possible)

(a) Lack of time

(b) Lack of space in the curriculum

(c) Lack of training

(d) Lack of interest from staff

(e) Lack of materials/resources

(f) Others (please specify)

10. If planetary health is not part of your curriculum do you intend to introduce it in the coming years?

(a) Yes

(b) No

11. Do you think there is a link between planetary health and indigenous knowledge systems?

(a) Yes

(b) No

12. To what extent do you think that there is a link between planetary health and climate change?

(a) No association

(b) Slight association

(c) Moderate association

(d) Great association

13. What do you suggest as a way(s) to improve the uptake of planetary health both in practice and at Schools of Public Health?
